# Wearable Devices in Colorectal Surgery: A Scoping Review

**DOI:** 10.3390/cancers16132303

**Published:** 2024-06-22

**Authors:** Konstantinos Kavallieros, Lampros Karakozis, Romilly Hayward, Emmanuel Giannas, Lucio Selvaggi, Christos Kontovounisios

**Affiliations:** 1 Department of Surgery and Cancer, Imperial College London, London SW7 2BX, UK; konstantinos.kavallieros19@imperial.ac.uk (K.K.); lampros.karakozis17@imperial.ac.uk (L.K.); romilly.hayward19@imperial.ac.uk (R.H.); emmanouil.giannas18@imperial.ac.uk (E.G.); c.kontovounisios@imperial.ac.uk (C.K.); 2 Department of General Surgery, Chelsea and Westminster Hospital, London SW10 9NH, UK; 3 Department of Advanced Medical and Surgical Sciences, University of Campania “Luigi Vanvitelli”, 80138 Naples, Italy; 4 Department of Surgery, The Royal Marsden Hospital, London SW3 6JJ, UK; 5 2nd Surgical Department, Evangelismos General Hospital, 10676 Athens, Greece

**Keywords:** wearable devices, colorectal surgery, colorectal cancer, scoping review

## Abstract

**Simple Summary:**

This research explores how wearable devices can be used to monitor patients undergoing colorectal surgery. By reviewing existing studies, we found that wearable devices are used in various stages of surgery. Devices worn primarily before surgery focus on assessing patient physical status and preparation, while devices worn primarily after surgery are used to monitor surgical complications. The findings from 20 studies and 10 different wearable devices suggest that standardised use of these devices could lead to more proactive and personalised care, ultimately improving surgical outcomes and patient well-being.

**Abstract:**

Wearable devices are increasingly utilised to monitor patients perioperatively, allowing for continuous data collection and early complication detection. There is considerable variability in the types and usage settings of wearables, particularly within colorectal surgery. To address this, a scoping review was conducted to investigate current utilisation of wearable devices in colorectal surgery. A systematic search across MEDLINE and Embase was conducted following PRISMA Scoping Review guidelines. Results were synthesised narratively, categorised by perioperative phase (preoperative; postoperative; combination), and supplemented with descriptive statistics and tables. Out of 1525 studies initially identified, 20 were included, reporting data on 10 different wearable devices. Use of wearable devices varied across settings with those used preoperatively tending to focus on baseline physical status or prehabilitation, while postoperative use centred around monitoring and identification of complications. Wearable devices can enhance perioperative monitoring, enable proactive interventions, and promote personalised care for improved patient outcomes in colorectal surgery.

## 1. Introduction

Given an aging population, the demand for general surgical interventions is expected to increase [1]. This is best seen in the management of colorectal cancer, the third most prevalent cancer worldwide, where a combination of surgery and neoadjuvant chemotherapy have led to remarkable improvements in survival rates over the years [2,3]. This can largely be attributed to improvements in surgical technique and perioperative care. However, with growing demand, there is a need to optimise resource allocation; fundamental to this is patient risk stratification and accurate perioperative assessment, including detailed patient monitoring [4,5,6,7].

Digital health interventions in the form of wearable devices are increasingly used to improve monitoring of surgical patients [8]. They allow for the collection of continuous objective data in the patient’s natural environment and, in some cases, have been shown to detect complications earlier [8]. Wearable devices have been examined in various surgical specialties with promising results, often as part of an enhanced recovery program [5]. Che Bakri et al. demonstrated that wearables can be used as a valid tool to measure patient recovery following breast surgery and reconstruction [9,10]. There is considerable variability in the type and setting in which wearables are used in colorectal surgery. In this context, we designed a scoping review aiming to explore the literature and determine how wearable devices are currently being used in colorectal surgery patients, with classification of devices based on their use preoperatively, perioperatively, and postoperatively.

## 2. Methods

The following review is reported according to the PRISMA Scoping Review (ScR) guidelines [11].

### 2.1. Inclusion and Exclusion Criteria

Included studies were full-text papers in the English language of original research studies (randomised controlled trials, observational studies, cohort studies, case-control studies, validation studies), implementing a wearable technology device with patients undergoing colorectal surgery. Studies investigating the use of wearables throughout the patient journey (i.e., from preoperatively in the community, to perioperatively in the hospital, to postoperatively in the community) were included. Studies assessing multiple surgical specialties were included only if data were segregated such that the extraction of colorectal-specific outcomes was possible. 

Case reports, editorials, commentaries, and opinion pieces were excluded. Studies that did not explicitly describe data collected by the wearable device were also excluded. 

### 2.2. Search Strategy

A systematic search was performed in MEDLINE and Embase databases accessed via the OVID platform. A comprehensive search strategy was developed to maximise sensitivity (Appendix A), including key phrases for colorectal surgery and different terminology for wearable devices, combined with ‘AND’ and ‘OR’ Boolean operators. The references of chosen papers and relevant reviews were examined to ensure comprehensive inclusion of all relevant studies.

Titles and abstracts were screened by two reviewers independently. Subsequently, full texts of potentially eligible studies were retrieved and assessed. Discrepancies were resolved through discussion, with involvement of a third reviewer if consensus could not be reached. 

### 2.3. Charting the Data

A standardised data extraction form on the Covidence platform was used for data extraction. Data extracted included: author(s), year of publication, sample size, country of origin, study design, types of colorectal operations, details of the wearable device and parameters assessed, time of wearable device usage, results relevant to the device, and patient adherence. 

### 2.4. Data Synthesis

Results were collated and summarised in a narrative form, discussing the nature and distribution of the studies, and demographic factors. Wearable devices were categorised according to setting: preoperatively in the community; perioperatively in the hospital; and postoperatively in the community. Results were accompanied by tables summarising the main characteristics and findings of the included studies. Data were analysed using SPSS v.29 (IBM Corp., Armonk, NY, USA). Continuous variables were presented as median with interquartile range (IQR); discrete variables were presented as number and percentage. Figures were generated using PRISM Version 10.2.2 (Graphpad, San Diego, CA, USA).

## 3. Results

### 3.1. Study Demographics

**Figure 1:**
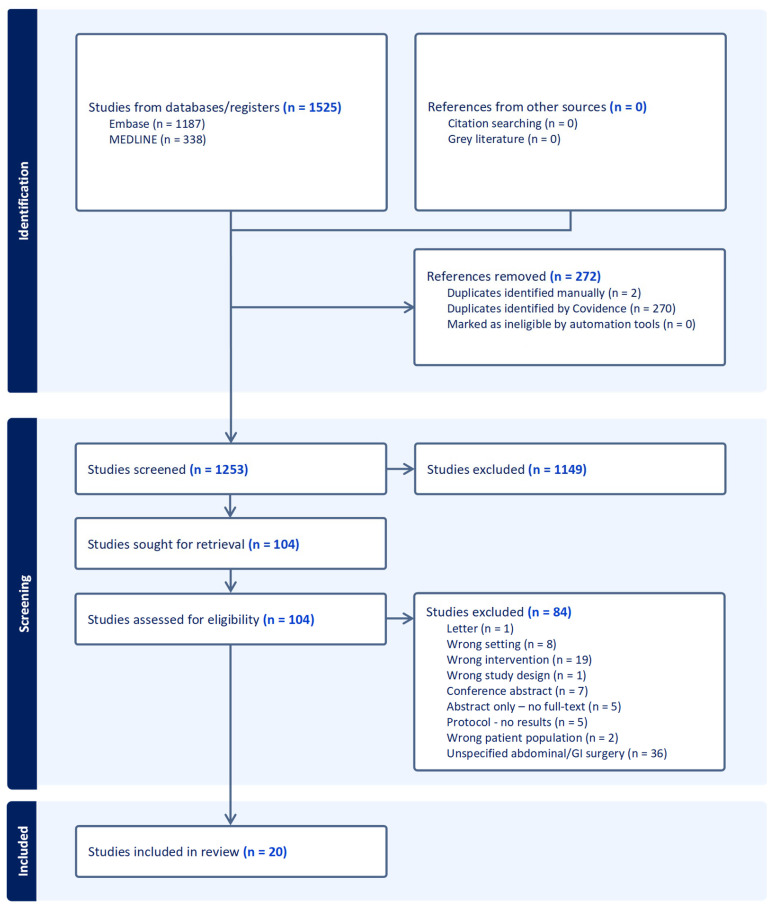
PRISMA flow-diagram of included studies.

The search strategy yielded a total of 1525 studies. Following deduplication, 1253 were screened, resulting in 104 studies for full text review, 20 of which were included in this review (Figure 1) [12,13,14,15,16,17,18,19,20,21,22,23,24,25,26,27,28,29,30,31]. Table 1 summarises key study characteristics. The median date of publication was 2021 (range: 2015–2024); the median number of participants was 65 (IQR: 29.0–99.5). Most studies were cohort (n = 16, 80%); with three (15%) randomised prospective and a type 2 hybrid design before–after study. The majority were published from European centres, (n = 9, 45%), with eight studies from the USA (40%), two studies from China and one from Lebanon.

**Figure 2:**
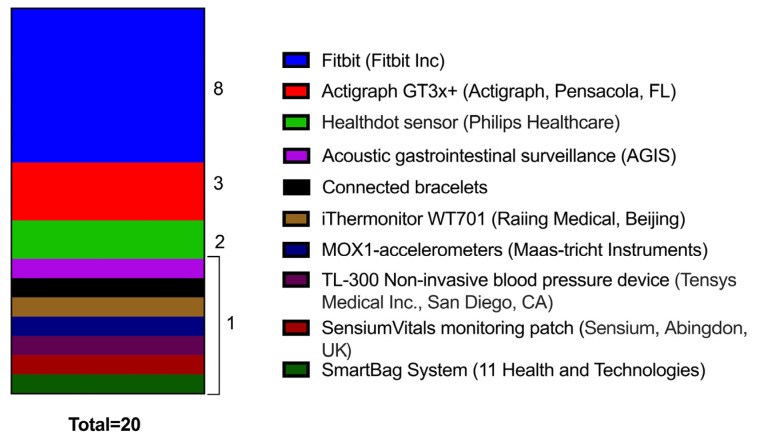
Names of wearable devices identified and number of studies utilising each device.

Three studies used wearable devices solely in the preoperative setting, seven in the postoperative setting, and the remaining across preoperative and postoperative settings (Table 2, Table 3 and Table 4). Ten different wearable devices were used: Fitbit (Fitbit Inc., San Francisco, CA, USA) (n = 8), Actigraph accelerometer (Actigraph, Pensacola, FL, USA) (n = 3), MOX1 accelerometer (Maastricht Instruments, Maastricht, The Netherlands) (n = 1), Healthdot sensor (Philips Healthcare, Best, The Netherlands) (n = 2), Sensium chest patch (Sensium, Abingdon, UK) (n = 1), Gastrointestinal biosensor (n = 1), SmartBag system (n = 1), Connected bracelets (n = 1), iThermonitor WT701 (Raiing Medical, Boston, MA, USA) (n = 1), and the TL-300 Blood pressure (BP) measurement device (n = 1) (Tensys Medical Inc., San Diego, CA, USA) (Figure 2).

### 3.2. Preoperative Data

Three studies analysed wearable devices in an entirely preoperative setting (Table 2). Two reported the data from a Fitbit Charge 2 assessing activity levels or sleep; the other employed the MOX1-accelerometer (attached to the thigh) to identify sedentary behaviour.

Waller et al. [13] randomised patients to standard care or prehabilitation comprising exercise and dietary advice delivered using a Fitbit connected to a smartphone application. Fitbits were worn by all participants for 3–4 weeks preoperatively to track physical activity. Authors reported increased daily physical activity levels (36.1 min vs. 17.5 min vigorous intensity, *p* = 0.022; 25.1 min vs. 13.1 min moderate intensity, *p* = 0.063) in the prehabilitation group, alongside improvements in 6-minute-walk test results from baseline to the day before surgery.

Kane et al. [26] used the same Fitbit device to study sleep prior to surgery in relation to 30-day postoperative outcomes. While sleep duration was not associated with postoperative outcomes, lower REM sleep duration correlated with increased complication risk and less than 1 h of REM sleep was associated with greater incidence of infectious complications and return to theatre. In contrast, very high nightly REM sleep duration (greater than around 2.5 h) was associated with increased risk of complications.

Finally, Argillander [16] et al. measured time spent in different postures and activities for 1 week prior to surgery. Authors correlated sedentary behaviour with frailty and worse physical outcomes as defined by a lower Short Physical Performance Battery score (*p* = 0.04), reduced hand grip strength (*p* = 0.03), lower Short-Form-36 score (*p* = 0.007), and lower motivation for movement (*p* = 0.02) and prehabilitation (*p* = 0.03). Additionally, sedentary patients had a significantly higher Fried frailty score > 3 (*p* = 0.03). 

### 3.3. Perioperative Data

Ten studies assessed the use of wearable devices in the perioperative period. Four studies utilised Fitbit devices to assess activity levels, two studies used the Actigraph GT3X accelerometer, and one study used the connected bracelets. Three studies assessed other physiological parameters including temperature via the iThermonitor (n = 1), motility activity via the acoustic gastrointestinal surveillance (AGIS) biosensor (n = 1), and Blood pressure (BP) via the TL-300 wearable (n = 1). 

Three studies utilised Fitbit devices to investigate the effect of physical activity on postoperative outcomes, including length of stay (LOS), postoperative complications, readmission rates, and postoperative activity. Daskivich et al. [21] demonstrated that step count up to 1000 steps on postoperative day 1 was associated with a lower odds ratio (OR) of extended LOS (OR 0.63, *p* = 0.003).

Hedrick et al. [22] demonstrated active patients with daily step count >5000 had significantly lower complication rates (n = 11 (27.5%) vs. n = 33 (55.9%); *p* = 0.005) and reduced incidence of serious complications (n = 2 (5%) vs. n = 12 (20.3%); *p* = 0.032). LOS was, however, similar between active and non-active patients (mean = 4.2 vs. 3.3 days, *p* = 0.070). Kane et al. [25] showed that return to baseline physical activity was significantly lower in patients who required readmission (median 15.1% vs. 31.8%; *p* = 0.004), with a 28.9% return to baseline physical activity identified as the threshold for readmission prediction (sensitivity 75.0%, specificity 74.0%). 

Van der Linder et al. [28] assessed the usability, acceptability, and feasibility of physical activity and food intake monitoring using the Fitbit device in patients undergoing colorectal surgery. Daily step count was recorded from 4 weeks preoperatively until 6 weeks postoperatively. Although not statistically significant, patients achieving >7500 daily steps and >1.2 g/kg protein intake tended to have reduced LOS (n = 4, 31% vs. n = 5, 50%, *p* = 0.42), complications (n = 1, 8% vs. n = 4, 40%, *p* = 0.13), and readmissions (n = 0, 0% vs. n = 1, 10%, *p* = 0.44). Overall, Fitbit Charge 3 had a median system usability score of 85/100 with 100% and 50% adherence in preoperative and postoperative measurements, respectively.

Two studies used the Actigraph GT3X accelerometer to measure postoperative activity in colorectal patients. Wilnerzon et al. [31] compared the effect of immediate mobilisation to standard care on postoperative physical activity. Authors did not demonstrate any statistically significant difference in postoperative physical activity between groups at any timepoint (adjusted mean ratio 0.97 on POD 1, *p* = 0.84; 0.89 on POD 2, *p* = 0.39; and 0.90 on POD 3, *p* = 0.44). 

Similarly, Wijma et al. [17] investigated the postoperative physical activity of major abdominal cancer surgery patients, including colorectal surgery, using the Actigraph GT3X. Significant correlations were identified between reduced postoperative physical activity and complication occurrence (OR 3.197, *p* = 0.039) and length of stay (β 4.068, *p* = 0.013). Another device used by Romain et al. [23], the connected bracelets, assessed preoperative and postoperative physical activity. Preoperative step count was significantly associated with increased postoperative physical activity (r = 0.527, *p* < 0.001).

Three studies used non-activity wearables to measure physiological parameters. Sun et al. [20] used the TL-300 non-invasive radial artery blood pressure (BP) measurement device, worn around the wrist, to investigate the accuracy of radial artery applanation tonometry (RAAT) compared to conventional arterial catheterisation blood pressure measurement in 30 colon cancer surgical cases. It was demonstrated that TL-300 may be an acceptable alternative to invasive BP monitoring, according to ANSI/AAMI standards.

Dai et al. [27] assessed intraoperative temperature measurement, comparing the accuracy of the iThermonitor wireless axillary sensor with a bladder probe in laparoscopic rectal surgery patients. They demonstrated iThermonitor to be significantly more accurate compared to the bladder probe and sufficiently accurate for intraoperative temperature measurement, with 95.21% of measurements being within ±0.5 °C of the reference core temperature, measured with a distal oesophageal probe.

Finally, Kaneshiro et al. [19] assessed the ability of a non-invasive acoustic gastrointestinal surveillance (AGIS) biosensor adhered to the abdominal wall to predict postoperative ileus (POI) by measuring motility events per minute. Motility events were recorded 60 min prior to surgery and continuously postoperatively. Authors reported the AGIS biosensor to be able to predict POI onset with 63% sensitivity, 72% specificity, 81% negative-predictive value, and, overall, >80% certainty.

### 3.4. Postoperative Data

Seven studies assessed wearable devices in an entirely postoperative setting. Two studies utilised Fitbit devices to access activity levels and/or sleep, while one used the Actigraph GT3+ accelerometer to record activity levels. Three studies monitored vital signs with a patch device (Sensium Vitals n = 1, Healthdot sensor n = 2), and one study utilised a SmartBag system to monitor ostomy data.

Skender et al. [18] assessed feasibility of physical activity monitoring with the Actigraph GT3+ accelerometer attached to an elastic belt worn below the chest. Of the eligible participants, 59% agreed to take part; 83% completed the assessment with at least 4 days of data. Step counts measured by accelerometery and pedometry were strongly correlated (ρ = 0.91, *p* < 0.0001).

Likewise, Yi et al. [12] demonstrated the utility of wearable biosensors in determining associations between physical activity, sleep quality, and postoperative LOS for inflammatory bowel disease patients. A shorter sleep duration and higher sleep efficacy were associated with shorter LOS. Furthermore, Allen et al. [15] used a Fitbit Inspire HR device to monitor sleep quality of postoperative patients. The percentage of patients achieving the CDC goal of seven or more hours per night ranged from only 10.9% on night 1 to 35.3% on night 3. They concluded that devices were able to capture severe sleep disturbance experienced by inpatients following surgery.

Downey et al. [14] compared standard intermittent National Early Warning Score (NEWS) monitoring with continuous monitoring of vital signs (CMVS) in an inpatient setting using the SensiumVitals remote monitoring patch worn on the chest. The patch monitored heart rate, respiratory rate, and temperature, and alerted the nursing team of deviations from physiological norms. This study reported on the feasibility of CMVS with the remote monitoring patch with a recruitment rate of 91.3% and a 71.7% rate of adherence to protocol. CMVS participants had fewer unplanned critical care admissions (1 versus 5) and shorter LOS (11.6 versus 16.2 days) with cost–utility analysis demonstrating the benefit of the remote monitoring system when compared to standard NEWS monitoring.

Leenen et al. [29,30] published two papers analysing the Healthdot sensor chest patch, which measured heart rate and respiratory rate. In 2023, authors reported the feasibility of a home monitoring system involving 5 days of CMVS via the sensor patch alongside three teleconsultations. There was a 68% agreement between CMVS measurements and telephone consultations. Patient satisfaction score for the home monitoring system was 8 out of 10 and the authors concluded that the monitoring system was feasible.

Subsequently, in 2024, authors reported a significant reduction in LOS for colorectal patients receiving CMVS in a postoperative inpatient setting when compared to standard care (4 vs. 4.5 days, *p* = 0.001). Though all other clinical outcomes were similar between groups, a non-significant trend towards less severe complications and reduced ICU LOS was noted in the CMVS group (3 vs. 8 days, *p* = 0.132). 

Finally, Fearn et al. [24] assessed the impact of a novel ‘SmartBag’ device, which allows real-time ostomy output, device usage, and skin condition monitoring, on hospital-based acute care resource utilisation for 30 days following ostomy surgery. The device comprised a monitoring ostomy appliance and skin wafer connected to a smartphone application, allowing real-time monitoring of ostomy output, device usage, and skin condition. Authors reported lower rates of hospital readmission (15.1% vs. 24.7%) and emergency department visits (6.1% vs. 17.7%) for patients using the SmartCare system compared with previously reported national benchmarking data.

## 4. Discussion

Our findings add to those of recent systematic reviews [8,32] assessing patient outcomes post-surgery, with a specific focus on colorectal surgery. Wearable devices integrated in preoperative and postoperative settings present a multifaceted approach to enhance patient care by facilitating prehabilitation interventions, predicting postoperative LOS, and potentially detecting complications early for timely interventions and optimised outcomes.

In the preoperative phase, studies used Fitbit devices and MOX1 accelerometers to monitor physical activity and sleep patterns [13,16,26]. Studies utilising Fitbit devices demonstrated correlations between preoperative and postoperative physical activity levels with various outcomes such as LOS, complication rates, and readmission rates in colorectal surgery patients. Moreover, implementing prehabilitation programs with wearables shows positive results in improving physical functioning before surgery [13]. Transitioning to the perioperative period, Fitbit devices showed some promise in monitoring recovery trajectories and predicting complications like LOS and readmission rates by tracking physical activity levels. These, in turn, were used to provide insights into patients’ recovery trajectories, enabling early intervention when necessary [21,22,25,26]. Additionally, non-invasive monitoring devices like connected bracelets for step-count measurement and the AGIS biosensors show potential in predicting postoperative complications, aiding in early identification and management [19,21]. The TL-300 for BP monitoring and the iThermonitor for temperature monitoring illustrate the potential to transition to wearable devices to measure physical parameters with accuracy intraoperatively [20,27].

Furthermore, the utilisation of wearable devices extends beyond traditional hospital settings into community-based care and specialised interventions. Studies conducted in both inpatient and community settings explored the feasibility and efficacy of wearable devices in monitoring postoperative recovery [12,14]. From monitoring sleep disturbances in inpatient settings to facilitating early discharge through home monitoring systems in community settings, wearable devices have the potential to offer versatile solutions for optimising postoperative care [15,29,30]. The integration of non-invasive monitoring tools such as the Healthdot sensor and the SmartBag system enabled remote monitoring and early intervention, thereby reducing hospital-based acute care utilisation [24,29]. 

The integration of wearables in colorectal surgery has significant potential for enhancing patient care and aiding surgical practice. By providing real-time feedback on vital signs and physical activity, wearables can enhance patient monitoring before, during, and after surgery, alerting medical professionals to any anomalies promptly. Furthermore, more research on wearables can strengthen already observed associations between physical activity and postoperative outcomes and complications, with the potential to devise statistical models to predict complications to allow early intervention and mitigate surgical risks. Moreover, wearables can facilitate remote patient monitoring, enabling healthcare providers to track patients’ recovery progress outside the hospital setting. Allowing patients to track their recovery and medical professionals to monitor them remotely has led to the concept of ‘virtual wards’, which could have important cost-saving implications for national health services [33,34]. However, there is a need for more evidence to support these claims [33,34].

It is important to note that despite the clear potential of the integration of wearable devices in the healthcare setting, there still exist significant limitations. Concerns are multifaceted and include uncertainties in data security and privacy and lack of industry standards, as well as general technological barriers [35]. More specifically, devices that can collect wide-ranging user information such as geographical location [36], and those which implement mobile linkage of the data, could risk data leakage and tampering; studies have reported user distrust of devices due to lack of understanding and other reasons, including the above reasons [37,38]. Furthermore, there is a need for concrete industry guidance and regulations to ensure adequacy of devices if they are to be widely implemented [39,40,41]. Finally, the devices identified in this review were lacking in integration of multiple functions in a single device; there is a need for technological advancement to expand the functions of individual devices and to allow greater device compatibility and versatility [42].

## 5. Limitations

While this scoping review provides valuable insights into the use of wearable devices in perioperative care, several limitations should be acknowledged. Firstly, the included studies vary in terms of methodology and outcome measures, which may limit the generalisability of their findings. In particular, the studies exhibit large variability in terms of study design. Included studies range from randomised controlled trials, observational studies, and cohort studies to validation studies. This variability makes comparisons of results more challenging due to each study presenting different levels of evidence. Additionally, a number of studies relied on qualitative descriptions of scores rather than quantitative comparisons, hindering the ability to conduct quantitative statistical comparisons to assess device utility. This qualitative approach limits the depth of understanding regarding the effectiveness of wearable devices in perioperative care and precludes the ability to draw definitive conclusions regarding their impact on patient outcomes. Furthermore, the heterogeneous nature of wearable devices and their applications across studies makes it challenging to standardise results or make direct comparisons between interventions. 

## 6. Conclusions

The incorporation of wearable devices in perioperative care presents a useful adjunct in the management of colorectal surgery patients. This technology provides valuable data for monitoring patient status and offers opportunities for proactive interventions to reduce postoperative complications by improving patients’ baseline physical activity prior to surgical intervention and predicting patients at higher risk of postoperative complications. As colorectal surgery develops, wearable devices have the potential to be a useful adjunct in perioperative care by providing personalised, real-time monitoring with accurate predictions of postoperative outcomes and intervention strategies tailored to individual patient needs.

## Figures and Tables

**Table 1:** Summary characteristics of included studies.

Author Name	Year	Country	Study Design	Total Number of Participants	Surgical Operations Included
Wijma 2023 [17]	2024	The Netherlands	Cohort study	143 (colorectal n = 28)	Hepatic resection n = 35 Colorectal resection n = 28 Pancreatic resection n = 55 Cytoreductive surgery; + hyperthermic intra-peritoneal chemotherapy n = 20 Non-therapeutic laparotomy n = 4 Other n = 1
Leenen 2024 [30]	2024	The Netherlands	Type 2 hybrid design before–after study	651	Colon n = 485 Rectal n = 166 HPB n = 257
Leenen 2023 [29]	2023	The Netherlands	Cohort study	21	Rt hemi n = 8 Sigmoid resection n = 6 APR n = 3 Wig resection n = 2 Ileocecal resection n = 1
Wilnerzon Thorn 2024 [31]	2023	Sweden	Randomised controlled trial	144	APR n = 17 other colon resection n = 1 Anterior resection rectum n = 17 Exploratory laparotomy n = 1 Ileocecal/rt hemi n = 40 Lt hemi n = 5 other large/SB procedure n = 4 other stoma procedure n = 29 Sigmoid resection n = 24 SB resection n = 5 Total/subtotal colectomy n = 3
Argillander 2022 [16]	2022	The Netherlands	Cohort study	47	Hemicolectomy, sigmoidectomy, lower anterior/abdominoperineal resection
Dai 2023 [27]	2022	China	Cohort study	82	Laparoscopic rectal surgery
van der Linden 2022 [28]	2022	The Netherlands	Cohort study	28	Colorectal carcinoma surgery, lap n = 27, open n = 1
Kane 2022 [26]	2022	United States	Cohort study	95	Colorectal surgery
Allen 2021 [15]	2021	Lebanon	Cohort study	64	Colectomy n = 45 other n = 19
Kane 2022 [25]	2021	United States	Cohort study	94	Rectal resection n = 27 Ostomy reversal n = 19 Right colon/ileocolic resection n = 16 Left colon/sigmoid resection n = 13, Total colectomy/proctocolectomy n = 7 other (SB resections, loop ostomy creations, and other intraabdominal procedures) n = 12
Waller 2020 [13]	2021	UK	Randomised controlled trial	22	Cytoreductive surgery and hyperthermic intraperitoneal chemotherapy n = 16 (n = 9 prehab, n = 7 control) APR n = 2 (control) Total pelvic clearance n = 2 (1 in each group) Rt hemi + cystectomy n = 1 (control) laparotomy + SB resection n = 1 (prehab)
Yi 2019 [12]	2021	United States	Cohort study	37	Stoma formation n = 15 Ileo-colectomy n = 14 total colectomy/proctocolectomy n = 5 Proctectomy n = 5 Subtotal colectomy n = 4 Abscess drainage n = 3 Fistula takedown n = 3 Ileostomy closure n = 3 Hartmann’s pouch n = 2 Lysis of adhesions n = 2 SB resection n = 2 Other n = 4 (stricturoplasty (1), excision of pouch (1), excision of Meckel diverticulum (1), and pelvic pouch repair)
Fearn 2020 [24]	2020	United States	Cohort study	66	Ostomy surgery
Downey 2020 [14]	2020	UK	Randomised controlled trial	136	Colonic resection (n = 108), SB resection (n = 4), other (n = 13)
Hedrick 2020 [22]	2020	United States	Cohort study	99	Elective colorectal surgery
Romain 2020 [23]	2020	Switzerland	Cohort study	50	Elective colorectal patients (except stoma closure) colon n = 38, rectum n = 7, other n = 5
Daskivich 2019 [21]	2019	United States	Cohort study	100	Open colectomy (n = 16), abdominal laparoscopic colectomy (n = 15) other (non-colorectal, n = 57)
Sun 2017 [20]	2016	China	Cohort study	30	Elective colon carcinoma surgery
Skender 2015 [18]	2015	United States	Cohort study	198	Colon/rectal cancer surgery
Kaneshiro 2016 [19]	2015	United States	Cohort study	28	SB n = 10 Partial colectomy n = 8 Total colectomy n = 4 Pelvic (APR) n = 5, other n = 1

HPB: Hepato-pancreato-biliary; Rt/Lt-hemi: right/left-hemicolectomy; APR: abdominoperineal resection; SB: small bowel; lap: laparoscopic; prehab: prehabilitation group.

**Table 2:** Devices used solely in the preoperative setting.

Study	Wearable Device	Parameters Assessed	Results	Adherence
Waller 2020 [13]	Fitbit Charge 2 (Fitbit Inc.)	Heart rate and step count. **Derived**: physical activity levels.	Prehabilitation group had more daily minutes of moderate and vigorous physical activity and greater improvements in 6 min walk test distance from baseline to the day before surgery when compared with controls.	Prehabilitation group participants wore the Fitbit on 98.9% of days.
Kane 2022 [26]	Fitbit Charge 2 (Fitbit Inc.)	Actigraphy (wrist movement), photoplethysmography (heart rate). **Derived**: sleep data.	Total sleep duration was not associated with any postoperative outcomes. Lower REM sleep duration correlated with increased complication risk. Very high nightly REM sleep duration was associated with increased risk of complication development.	Not reported
Argillander 2022 [16]	MOX1-accelerometer (Maastricht Instruments, Maastricht, The Netherlands)	Raw acceleration data. **Derived**: time spent in different postures (sitting/lying or upright) and activities (dynamic versus non-dynamic).	Preoperative sedentary behaviour was associated with frailty and worse physical functioning irrespective of cancer type.	n = 4 had less than 72 h of data and were excluded. n = 1 dropout. n = 7 excluded due to sensor/program malfunction/ missing sensors.

**Table 3:** Devices used both preoperatively and postoperatively.

Study	Wearable Device	Parameters Assessed	Results	Adherence
Kaneshiro 2016 [19]	Acoustic gastrointestinal surveillance (AGIS) biosensor	Intestinal motility	AGIS predicted Post Operative Ileus (POI) onset with 63% sensitivity, 72% specificity, 81% NPV, and over 80% accuracy.	Not reported
Sun 2017 [20]	TL-300 non-invasive radial artery blood pressure measurement device (Tensys Medical Inc., San Diego, CA, USA)	Blood pressure	BP measurements obtained via the TL-300 had clinically acceptable agreement (as defined by the AAMI criteria) with data that were acquired invasively using an arterial catheter.	Not reported
Daskivich 2019 [21]	Fitbit Charge (Fitbit Inc.)	Step count	Step count up to 1000 steps on postoperative day 1 was associated with lower probability of a prolonged LOS.	n = 15 excluded owing to loss of activity monitor and data
Hedrick 2020 [22]	Fitbit Charge 2 (Fitbit Inc.)	Heart Rate; Step count	Active patients with daily step count > 5000 had significantly lower complication rates and reduced incidence of serious complications. LOS was, however, similar between active and non-active patients.	n = 7 patients experienced technical problems with the Fitbit or did not return it, resulting in a lack of postoperative data
Romain 2020 [23]	Connected bracelets	Step count	The number of preoperative footsteps was significantly correlated to postoperative physical activity.	Not reported
Kane 2022 [25]	Fitbit Charge 2 (Fitbit Inc.)	Heart Rate; Step count	Return to baseline physical activity was significantly lower in patients who required readmission with a 28.9% return to baseline physical activity identified as the threshold for readmission prediction.	n = 12 experienced technical problems with the wearable device or did not return it resulting in a lack of postoperative data
Dai 2023 [27]	iThermonitor WT701 (Raiing Medical, Boston, MA, USA)	Axillary Temperature	95.21% of iThermonitor temperature measurements were within ±0.5 degrees Celsius of the reference core temperature during the whole surgical period.	100% adherence
van der Linden 2022 [28]	Fitbit Charge 3 (Fitbit Inc.)	Step count	Median system usability score of 85/100, and +65 preoperative and +67 postoperative net promotor score (range −100 to +100) for acceptability.	100% adherence in preoperative measurements. Only 14 (50%) participants provided data on physical activity in all postoperative weeks timepoints
Wilnerzon Thorn 2024 [31]	Actigraph GT3X (Actigraph, Pensacola, FL, USA)	Step count; Accelerometer	No statistically significant difference in postoperative physical activity between groups at any of the measurement timepoints.	Accelerometer data not available for n = 9 preop, n = 9 for pod 1–3 and n = 13 for pod30
Wijma 2023 [17]	Actigraph GT3X (Actigraph, Pensacola, FL, USA)	Step count	Significant correlations were identified between reduced postoperative physical activity and complication occurrence and length of stay.	Data available for n = 143 for pod 1–4, n = 129 for pod 5, n = 117 for pod 6 and n = 104 for pod7

**Table 4:** Devices used solely in the postoperative setting.

Author Name	Wearable Device	Parameters Assessed	Results	Adherence
Skender 2015 [18]	Actigraph GT3x+ (Actigraph, Pensacola, FL, USA)	Raw acceleration data, activity counts, step counts, energy expenditure, sleep duration	Feasibility of accelerometery for assessing postoperative physical activity in colorectal cancer patients, independent of gender, tumour stage or BMI. 83% of the physical activity measurements were completed with data from at least 4 consecutive days.	6-months follow-up 54%; 12-months follow-up 48%; 24-months follow-up 46%
Yi 2019 [12]	Fitbit Charge or Alta HR (Fitbit Inc.)	Step count, sleep duration and efficiency (sleep:time in bed)	Demonstrated the utility of wearable biosensors in characterising associations between physical activity, sleep quality, and length of stay.	82.7% for step data; 80.1% for sleep data
Fearn 2020 [24]	SmartBag System (11 Health and Technologies, Irvine, CA, USA)	Ostomy output, device usage, skin condition	Hospital-based acute care resource utilisation < 30 days after ostomy surgery was lower for patients using SmartCare support system (hospital readmission 15.1% vs. 24.7%; ED visits 6.1% vs. 17.7%).	not reported
Downey 2020 [14]	SensiumVitals remote monitoring patch (Sensium, Abingdon, UK)	Heart rate, respiratory rate, temperature	Confirmed feasibility of the remote monitoring patch. Recruitment rate 91.3% of those eligible, adherence to protocol 71.7%.	From the intervention arm, 17 participants (28.3%) did not adhere to protocol due to discomfort/skin reaction (n = 5), too many false alerts (n = 2), transfer to non-participating ward (n = 1), and incorrect assumption of imminent discharge (n = 1).
Allen 2021 [15]	Fitbit Inspire HR (Fitbit Inc.)	Sleep	Percentage of patients achieving the CDC goal of 7+ h: night 1: 10.9%, night 2: 32.8%, night 3: 35.3%, night 4: 27.6%. Overall, 67 of 168 recorded nights (39.9%) had total sleep time of 7 h or more.	Research staff ensured compliance with wearing the device.
Leenen 2023 [29]	Healthdot sensor (Philips Healthcare)	Heart rate, respiratory rate	The intervention performance and patient acceptability were high, though the quality of vital sign data was variable.	not reported
Leenen 2024 [30]	Healthdot sensor (Philips Healthcare)	Heart rate, respiratory rate	Continuous monitoring of vital signs (CMVS) using wearable wireless sensors and proactive trend assessments was associated with a significant decrease in length of stay for colorectal surgery patients compared to patients receiving standard care.	not reported

## Data Availability

The data presented in this study are available in this article.

## Appendix A

Search string (Embase)

1. (Colorectal* OR MeSH Colon OR Colon* OR MeSH rectum OR rectum OR rectal* OR MeSH intestine OR bowel* OR MeSH colectomy OR Colectomy OR (abdominal adj3 (surgery or operation)) OR MeSH colorectal surgery
2. (MeSH surgery OR surgery OR MeSH Operation OR Operation))
3. 1 AND 2
4. Wearable* OR acceleromet* OR pedomet* OR “activity monitor*” OR “activity tracker*” OR “fitness tracker*” OR gyroscop* OR biosens*
5. Fitbit* OR “apple watch*” OR “smart watch*”
6. (Non-invasiv* OR wireless OR electronic or portable or digital or handheld or remote) adj3 (sens* OR monitor* OR track* or device*)
7. 3 AND (4 OR 5 OR 6)
